# Mechanistic Studies on the Use of *Polygonum multiflorum* for the Treatment of Hair Graying

**DOI:** 10.1155/2015/651048

**Published:** 2015-11-10

**Authors:** Ming-Nuan Han, Jian-Mei Lu, Guang-Yuan Zhang, Jie Yu, Rong-Hua Zhao

**Affiliations:** Yunnan University of Traditional Chinese Medicine, Kunming, Yunnan 650500, China

## Abstract

*Polygonum multiflorum* is a traditional Chinese medicine with a long history in hair growth promotion and hair blackening. The purpose of the study was to examine the effect and the mechanism of *Polygonum multiflorum* in hair blackening. C57BL/6 mice hair fade was induced with H_2_O_2_ and used in this research. Hair pigmentogenesis promotion activities of Polygonum Multiflorum Radix (PMR, raw crude drug), Polygonum Multiflorum Radix Preparata (PMRP, processed crude drug), and their major chemical constituent TSG were investigated. The regulation effects of several cytokines and enzymes such as POMC, *α*-MSH, MC1R, ASIP, MITF, TYR, TRP-1, and TRP-2 were investigated. PMR group gave out the most outstanding black hair among all groups with the highest contents of total melanin, *α*-MSH, MC1R, and TYR. Promotion of hair pigmentogenesis was slightly decreased after processing in the PMRP group. TSG as the major constituent of PMR showed weaker hair color regulation effects than both PMR and PMRP. PMR, but not PMRP, should be used to blacken hair. The *α*-MSH, MC1R, and TYR were the major targets in the medicinal use of PMR in hair graying. Chemical constituents other than TSG may contribute to the hair color regulation activity of PMR.

## 1. Introduction

Hair color is instantly recognized during human interactions, and hair graying is a sign of old age, ill health, and bodily decline, especially in Asian countries where black is the most common hair color. There is a strong desire to be young and vital, and thus premature graying has attracted many researchers. At the same time, the pharmaceutical or functional food industries are seeking targets for the prevention and treatment of hair graying. Despite the variety of products available with differing results and efficiencies, a completely satisfactory solution for hair graying remains incomplete.

**Figure 1:**
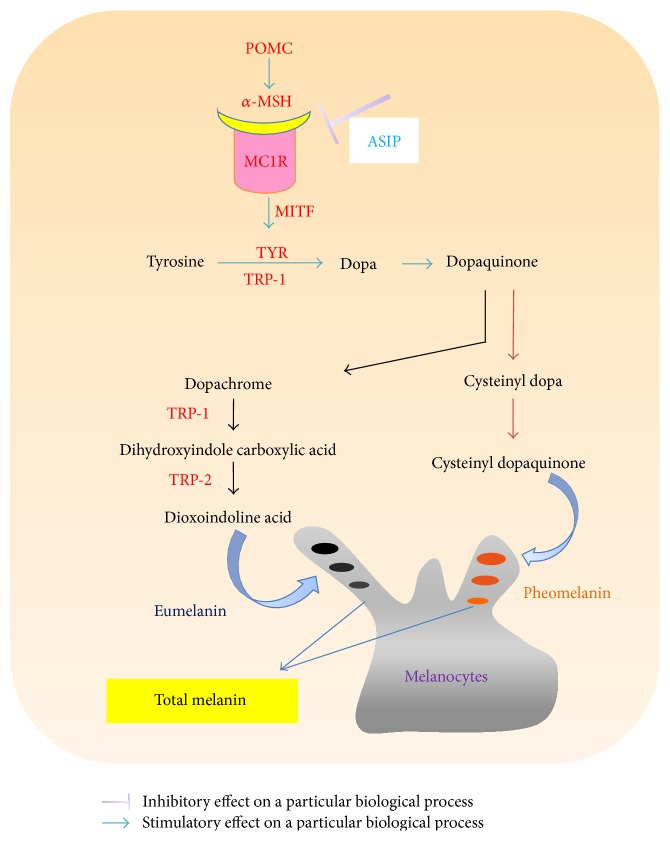
Major proteins and factors involved in the hair pigmentation.

Conditions that affect hair color include aging, achromotrichia [1], stress [2], medical conditions [3], and artificial factors [4]. Hair color is not only affected by genetic, age, and environmental factors but also affected by many cytokines and proteins. The generation of melanin is mainly regulated by POMC (proopiomelanocortin), *α*-MSH (*α*-melanocyte-stimulating hormone), MC1R (melanocortin 1 receptor), ASIP (agouti signaling protein), MITF (microphthalmia-associated transcription factor), TYR (tyrosinase), TRP-1 (tyrosinase related protein 1), and TRP-2 (tyrosinase related protein 2) (Figure 1).

In traditional Chinese medicine, preparations from* Polygonum multiflorum* have a long history of use for hair growth and blackening. Both oral and topical administration of* P. multiflorum* preparations were clinically used in the treatment of hair graying sometimes simultaneously in traditional application methods. RPM (Polygonum Multiflorum Radix, raw crude drug) and RPMP (Polygonum Multiflorum Radix Preparata, processed crude drug) were thought to be herbs that tonify the kidney and liver in traditional Chinese medicine theory, so that they could be used in the treatment of early graying of hair. Double blind, placebo-controlled studies lasting over 6 months have demonstrated marked beneficial effects of PMR on hair quality for pre- and postmenopausal women [5]. After taking extracts of* P. multiflorum* for 3 and 6 months, the surveys administered to the active treatment revealed a significant improvement of hair loss (25 in 26 participants, 97%) and perceived hair appearance (20 in 26 participants, %). Additionally, 77% of women in the* P. multiflorum* group reported “thicker hair,” which was rated as “significant” and “dramatic” improvement [5]. In another study employing 48, 30–60-year-old men (*n* = 24) and women (*n* = 24) with differing origins of hair loss (age-related, stress- and medication induced, and postpartum) received a standardized extract of PMR twice daily [6]. After 1 month of treatment, 91% of men and 87% of women reported improvement. Additionally, none of the study participants reported any side effects during the treatment period. However, the mechanisms by which PMR and PMRP exhibit these beneficial effects have yet to be elucidated.

We have studied* P. multiflorum* for decades. In our previous research [7], we found that both oral administration of PMR and topical administration of PMRP promoted hair growth. PMR was more suitable for oral administration, while PMRP showed greater effects in external use. The hair growth promotion effect of oral PMR was most likely mediated by the expression of fibroblast growth factor-7 (FGF-7). In this research, C57BL/6 mice hair fade induced by H_2_O_2_ was used to investigate hair pigmentogenesis promotion activity and a possible mechanism of PMR and PMRP. Their extractions were administered orally and/or topically. Hair pigmentogenesis promotion activities were investigated by hair color and total melanin contents. The regulation effects of several cytokines and enzymes such as POMC, *α*-MSH, MC1R, ASIP, MITF, tyrosinase, TRP-1, and TRP-2 were studied here.

## 2. Materials and Methods

### 2.1. Plants Materials and Chemicals

**Figure 2:**
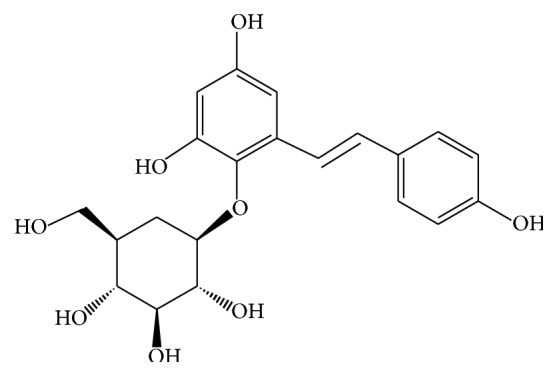
Structure of TSG.

The root of* Polygonum multiflorum* Thunb. were collected in June 2012 by the authors in Luda Rode, Pingshan Town, Luquan County, Yunnan Province and identified by Professor Ronghua Zhao of Yunnan University who is a specialist in traditional Chinese medicine (Specimen number: HMN 20120605). Voucher specimens were deposited in the Herbarium of Pharmacognosy, Yunnan University of Traditional Chinese Medicine. TSG (Figure 2) was purchased from Nanjing Jingzhu Bio-Technology Co., Ltd., China. The purity was over 98% via a high performance liquid chromatography-diode array detector.

### 2.2. Processing and Extraction of PMR and PMRP

PMRP was steamed from PMR with black soybean decoction according to the procedure recorded in Pharmacopoeia of the People's Republic of China (2010 edition) [8]. Black soybean decoction (25 kg) was obtained from 10 kg black soybeans boiled with water for two times (4 h for the first time and 3 h for the second time). 25 kg Black soybean decoction was needed in the steaming of 100 kg PRM.

**Figure 3:**
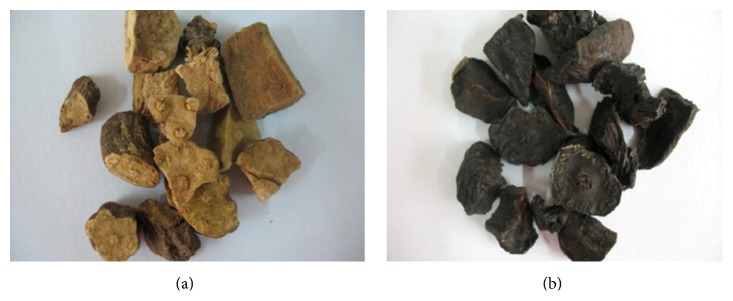
Photographs of raw (a) and processed (b) Polygoni Multiflori Radix.

The 300 g of finely ground PMR and PMRP (Figure 3) powder was successively extracted with 3, 2.4, and 1.8 L of water. Extracts were then combined and lyophilized.

### 2.3. HPLC-DAD Analysis of PMR and PMRP

All experiments were performed with a Dionex Ultimate 3000 HPLC system (Dionex Technologies, USA). Data were analyzed with Chromeleon 6.8.

The separations of RPM and RPMP extractions were achieved on a Zorbax SB-C18 analytical column (4.6 mm × 250 mm, ID, 5 *μ*m particle diameter from Agilent Technologies, USA).

The gradient elution used a mobile phase consisting of (A) 0.1% H_3_PO_4_ and (B) methanol. The following gradient program was used: 40% B (at the start), 70% (5 min), 80% (10 min), 85% (15 min), 90% (20–25 min), and then back to 40% (35 min). The detection wavelength was 254 nm. The oven temperature was 30°C, and the flow rate was 1.0 mL·min^−1^.

References standards of TSG were accurately weighed and dissolved in methanol. Extracts of PMR and PMRP were both weighted accurately and dissolved in 10 mL of 50% methanol. A 10 *μ*L injection was used for all analyses.

### 2.4. Animals and Treatments

Fifty six-week-old C57BL/6 male mice (weighed 20 ± 2 g) were provided by Beijing HFK Bioscience Co., Ltd. They were housed ten per cage in stainless steel cages containing sterile paddy husk as bedding in ventilated animal rooms (temperature 22 ± 1°C; 60 ± 10% humidity; and a 12 h/12 h light/dark cycle) with free access to water and a commercial laboratory complete food. All animal experiments were performed in compliance with the animal experimental ethics committee of Yunnan University of Traditional Chinese Medicine (R-062014025). All reasonable efforts were made to minimize the animals' suffering.

The mice were randomly assigned to 5 groups (*n* = 10) (Table 1) after adaptive feeding for three days. Group A was the control group with physiological saline given orally. Group B had nothing other than 0.0375% H_2_O_2_ solution on its back fur for six consecutive weeks. This served as the model group. The 0.0375% H_2_O_2_ solution was spread with a cotton swab on back fur of all mice except for the control group every morning. Six hours later, all mice were fasted for 2 h. Then, TSG (0.0034 g/mL in water), PMR (0.0576 g/mL in water), and PMRP (0.0576 g/mL in water) were given by gavage to mice in groups C, D, and E (0.2 mL/20 g). In the meantime, TSG (1.36 mg/mL in water), PMR (23.04 mg/mL in water), and PMRP (23.04 mg/mL in water) were rubbed in with a cotton swab on the back fur for topical given. The doses of PMR and PMRP were conversed from the recommended doses of RPM and RPMP in the Pharmacopoeia of the People's Republic of China, 2010 edition [8]. The dose of TSG was calculated from its concentration in PMR. No appropriate positive drug was used because there is no approved therapy for hair graying.

### 2.5. Investigation of Hair Growth Color Regulation Activities

Hair samples of all mice were collected of the same group on the last day of the experiment. All collected hair samples in the group were immediately combined, vortexed, and washed with 20 mL ddH_2_O twice to prevent color interference from the extracts or compounds. Then, 10 mg of hair were weighed and placed in glass test tube and 1 mL water and 9 mL organic solvent Soluene-350 (Soluene-350, a strong organic solvent that could dissolve a variety of tissues such as hairs) were added to each test tube [9]. The mixtures were heated twice for 45 min, with a short cooling period in between. The absorbance of the supernatants at 500 nm was measured for three times with a UV-Vis spectrophotometer (UV-4802H, Unico Instrument Co. Ltd., Shanghai).

### 2.6. Assessment of Related Proteins and Growth Factors in Skin

Mice skin was collected at the end on the last day of the experiment. Skin samples were disinfected with 75% ethanol and transferred to liquid nitrogen for storage after depositing immediately in −80°C for 24 h and then skin samples were weighed and washed with 0.9% saline before use. 100 mg skin samples in each group were cut into pieces and homogenated on ice with 1 mL physiological saline. The homogenates were centrifuged for 10 min at 4°C. The POMC, *α*-MSH, MC1R, MITF, ASIP, TYR, TRP-1, and TRP-2 contents in the supernatant were measured by Elisa kits (Cusabio Biotech Co., Ltd., and Bio-Swamp Life Science Lab).

### 2.7. Statistical Analysis

All data were expressed in the form of *X* ± SD. The data were evaluated by one-way analysis of variance (ANOVA) when multiple group comparisons were performed. Relationships between variables were assessed with Pearson's correlation coefficient.

## 3. Results

### 3.1. TSG Content in Tested Preparations

**Figure 4:**
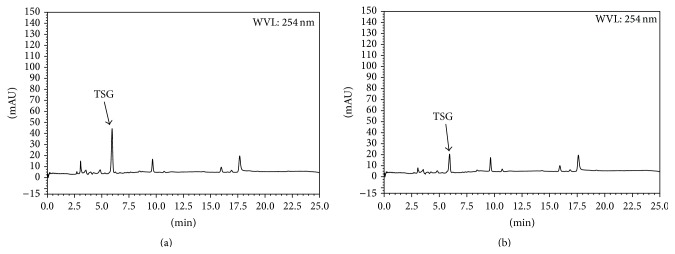
HLPC profiles of PMR (a) and PMRP (b).

Figure 4 shows HPLC-UV profiles of water extracts of PMR and PMRP. The peak eluting at 6.0 minutes was identified as TSG by comparison of the retention time and UV spectra with authentic standards. Linear relationships between the injected amount and the peak area were observed. The TSG concentration in PMR extract was 59.94. However, its concentration reduced to 29.36 mg/g in PMRP after processing. Because it was an abundant constituent of both PMR and PMRP [10], we tested this compound individually.

### 3.2. Hair Color Regulation Effects

**Figure 5:**
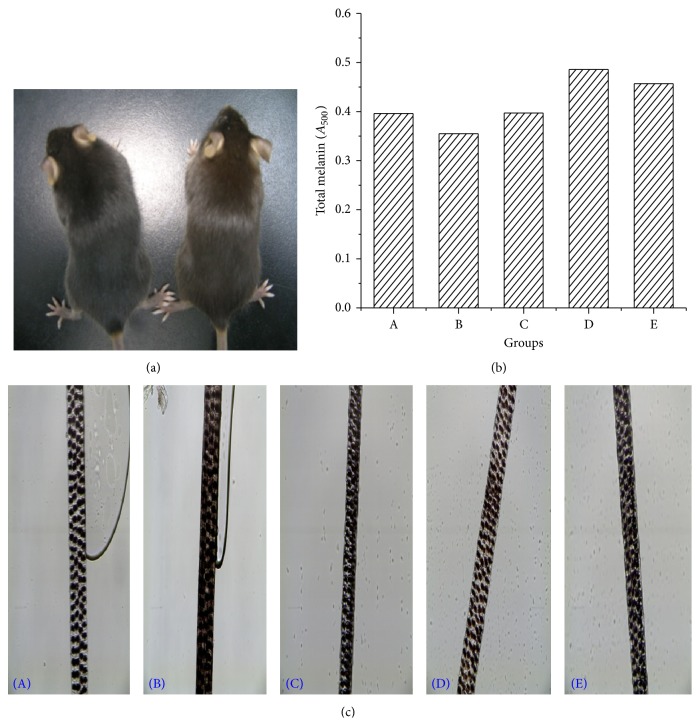
Hair color (a), total melanin contents (b), and microscopic images (c) of mice hair at the end of the research. (a) Mice appearances in control (left) and model (right) group. Obvious decolorization was observed in mice hair after H_2_O_2_ treatment. (b) Average total melanin content in a group was measured with a UV-Vis spectrophotometer. (c) Photomicrographs of mice hair in all groups demonstrate their color alteration. Mice were in control (A) or untreated (B), TSG (0.034 g/kg and 0.068 g/kg, oral and topical) (C), PMR (0.576 g/kg and 1.152 g/kg, oral and topical) (D), and PMRP (0.576 g/kg and 1.152 g/kg, oral and topical) (E) groups.

Obvious decolorization was observed in mice hair after H_2_O_2_ treatment (Figure 5(a)). Mice hair in the model group was a red-brown color but pure black in the control group mice. The total melanin content was significantly lower in the model group after fading with H_2_O_2_ (Figure 5(b))_._ However, this fading could be reversed by treatment with PMR and PMRP. The total melanin contents were 36.9% and 28.4% higher in the PMR and PMRP group than in the model group.

Photomicrographs of mice hair in all groups demonstrate their color alteration (Figure 5(c)). The red-brown mixed with black could be observed only in model group, while all other groups were darker.

### 3.3. Hair Color Regulation Mechanisms

Concentrations of hair color-related proteins and factors could be correlated directly to the generation of melanin. Therefore, we studied major proteins and factors involved in hair pigmentation.

**Figure 6:**
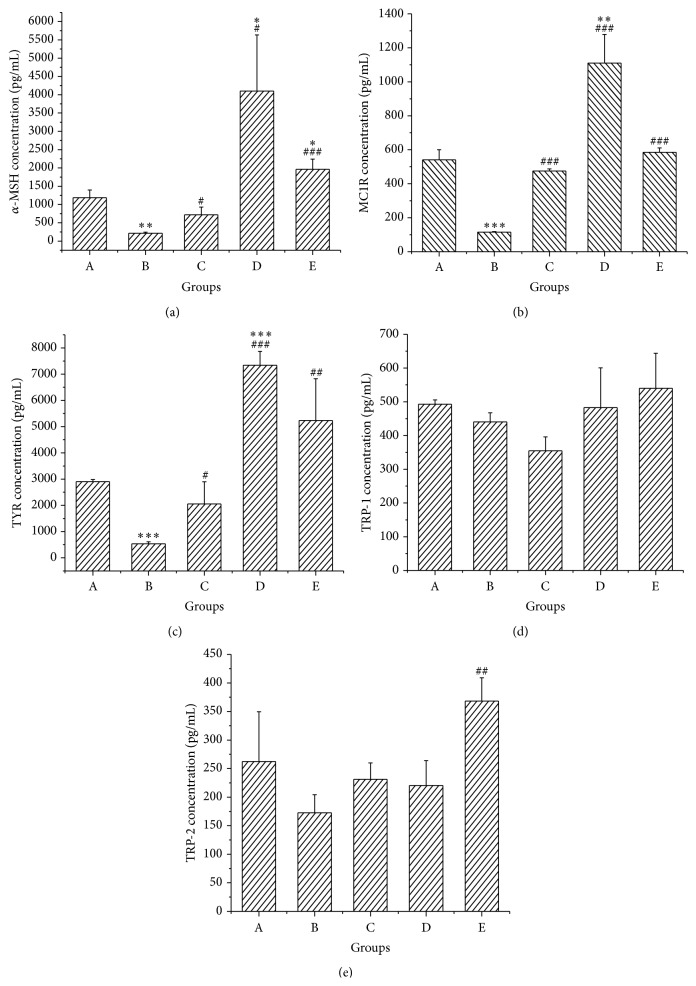
*α*-MSH (a), MC1R (b), TYR (c), TRP-1 (d), and TRP-2 (e) concentrations in skins of different groups. (X ± SD, *n* = 10). Mice were in control (A) or untreated (B), TSG (0.034 g/kg and 0.068 g/kg, oral and topical) (C), PMR (0.576 g/kg and 1.152 g/kg, oral and topical) (D), and PMRP (0.576 g/kg and 1.152 g/kg, oral and topical) (E) groups. The *α*-MSH, MC1R, TYR, TRP-1, and TRP-2 contents in the skin tissue were measured by Elisa kits. The # indicates a significant difference compared with untreated hair graying group. ^#^ *p* < 0.05; ^##^ *p* < 0.01; ^###^ *p* < 0.001. The *∗* indicates a significant difference compared with control group. ^*∗*^ *p* < 0.05; ^*∗∗*^ *p* < 0.01; ^*∗∗∗*^ *p* < 0.001.

Figures 6(a)–6(c) show that after treatment with the color fading reagent (0.0375% H_2_O_2_ solution) the expression of *α*-MSH, MC1R, and TYR decreased significantly and that all the treatments could reverse this effect. We found that the effect of PMR was the most prominent. The expression of *α*-MSH, MC1R, and TYR in the PMR group increased by 18, 8, and 12 times versus the model group, which were even higher than the control group. The PMRP could also increase the expression of *α*-MSH, MC1R, and TYR; however, the effect was slightly weaker than the PMR.

Expression of TRP-1 and TRP-2 proteins was not significantly affected by 0.0375% H_2_O_2_ solution (Figures 6(d) and 6(e)). Their changes after treatment were not obvious except that the expression of TRP-2 increased slightly in the PMRP group. No significant differences were observed in POMC, MITF, and ASIP among all groups (Figure S1 in Supplementary Material available online at http://dx.doi.org/10.1155/2015/651048).

### 3.4. Relationship Analysis between Total Melanin and Protein Expression

The relationships between total melanin contents and expressions of *α*-MSH, MC1R, TYR, TRP-1, and TRP-2 were presented in Table 2. As expected, the total melanin contents were positively correlated with the expression of *α*-MSH (*r* = 0.936, *p* < 0.05), MC1R (*r* = 0.9155, *p* < 0.05), and TYR (*r* = 0.989, *p* < 0.01). The PMR group had the most outstanding black hair among all groups with the highest total melanin content. At the same time, the expressions of *α*-MSH, MC1R, and TYR after treatment with PMR increased to levels even higher than before H_2_O_2_-induced fading.

## 4. Discussions

Human hair color is regulated by multiple factors (Figure 1), such as activation of the* POMC* gene [11, 12], expressions of *α*-MSH [13, 14], binding activities of *α*-MSH to MC1R [15], agonists of MITF, and antagonists of ASIP [16, 17]. Downregulation of TYR activity, which is the rate-limiting enzyme for controlling the production of melanin [18, 19], has been proposed to be responsible for reduced melanin production. In addition, expression or activity variations of TRP-1 and TRP-2 are eventually affecting the production of melanin [20–22]. However, underlying molecular and biochemical mechanisms of graying remain under debate. Therefore, there is no positive control drug because there is no approved therapy for hair graying.

Apart from various hair dyes of varying efficacy and duration, fully satisfactory solutions for the graying problem remain to be brought to market. Therefore, searching for effective and safe hair graying prevention and treatment drugs from traditional Chinese medicine or natural products has enormous social and economic benefits.

Recent* in vivo* research indicated that human gray/white scalp hair shafts accumulate hydrogen peroxide (H_2_O_2_) in millimolar concentrations. FT-Raman spectra showed* in vivo* the presence of 10^−3^ mol/L H_2_O_2_ concentrations in gray and completely white hair. The* in vivo* identification of massive H_2_O_2_ concentrations (determined by FT-Raman spectroscopy) in the gray hair shaft introduced a new step in the understanding of human hair graying on the biochemical and molecular biological level [23]. Therefore, C57BL/6 mice hair fade induced by H_2_O_2_ was used in this research in order to simulate human hair gray status.

In this research, our results provided justification for the traditional use of* P. multiflorum* against hair graying. We found that treatment with PMR could completely reverse the hair decolorization induced by H_2_O_2_. The PMR group had the most outstanding black hair among all groups with the highest content of total melanin, *α*-MSH, MC1R, and TYR. The *α*-MSH, MC1R, and TYR were the major targets for the medicinal use of PMR in hair graying. *α*-MSH, MC1R, and TYR were all critical factors that affect the formation of melanin from tyrosine via dopa (Figure 1). However, these effects were weaker in the PMRP group. Therefore, PMR should be used for hair blackening, but not PMRP.

The doses of PMR and PMRP in this research were conversed from the recommended doses of RPM and RPMP in the Pharmacopoeia of the People's Republic of China, 2010 edition. Fortunately, there was no adverse effect reports under these recommended doses. Therefore, the doses used in our research were considered to be safe.

TSG showed hair color regulation effects that were weaker than both PMR and PMRP. As we mentioned before, TSG is major chemical constituent of* P. multiflorum* before and after processing. We conclude that TSG might partly contribute to hair color regulation and that constituents other than TSG are responsible for the hair color regulation activity of PMR. The active chemical constituent(s) should be identified in future work; this may be a promising area of future research. Meanwhile, whether oral or topical application is more important should also be clarified in the future research.

## 5. Conclusion

Rationality of traditional use of* P. multiflorum* against hair graying from ancient times is provided in this research. Extracts of* P. multiflorum*, especially from raw crude drug, could completely reverse the hair decolorization induced by H_2_O_2_. Expressions of *α*-MSH, MC1R, and TYR are upregulated and then more melanin is produced as a consequence. In our research, PMR, but not PMRP, showed better effect on hair blackening. However, further clinical investigations were still needed to provide more solid and scientific evidences.

## Supplementary Material

Expression of POMC, MITF and ASIP proteins was not significantly affected by 0.0375% H_2_O_2_ solution (Figure S1(a)-(c)) and their changes were not obvious after treatment.

## Figures and Tables

**Table 1:** Animal grouping and treatments.

Groups	Hair color fading reagent	Treatments	Drug-delivery route	
			and dosage (g/kg)	
			Oral	Topical
A	—	Physiological saline	—	—
B	H_2_O_2_	Physiological saline	—	—
C	H_2_O_2_	TSG	0.034	0.068
D	H_2_O_2_	PMR	0.576	1.152
E	H_2_O_2_	PMRP	0.576	1.152

**Table 2:** Pearson's correlation coefficients between total melanin and related proteins and enzymes.

	*α*-MSH	MC1R	TYR	TRP-1	TRP-2

Total melanin	*r* = 0.936^*∗*^	*r* = 0.9155^*∗*^	*r* = 0.989^*∗∗*^	—	—

^*∗*^ *p* < 0.05; ^*∗∗*^ *p* < 0.01.
