# Supplementary material for: Mechanistic Studies on the Use of Polygonum multiflorum for the Treatment of Hair Graying
Source: Biomed Res Int. 2015 Nov 10;2015:651048. doi: 10.1155/2015/651048 (PMC4657090; doi:10.1155/2015/651048)
Supplement: Supplementary file 1 — Expression of POMC, MITF and ASIP proteins was not significantly affected by 0.0375% H2O2 solution (Figure S1(a)-(c)) and their changes were not obvious after treatment. [file 651048.f1.pdf]

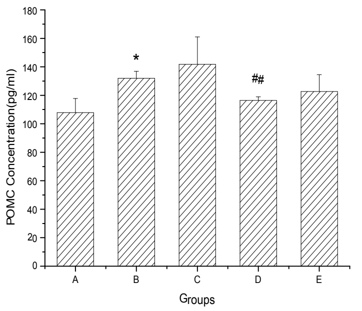

(I)

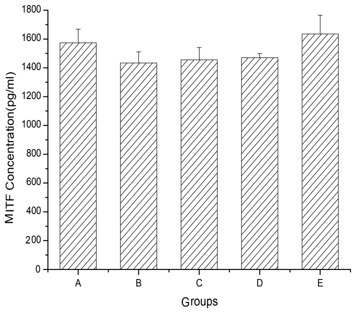

(II)

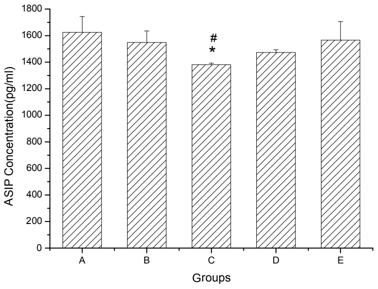

(III)

Figure S1: POMC (I), MITF (II) and ASIP (III) concentrations in skins of different groups. (X±SD, n=10)

Mice were in control (A) or untreated (B), TSG (0.034 g/kg and 0.068 g/kg, Oral and Topical) (C), PMR (0.576 g/kg and 1.152 g/kg, Oral and Topical) (D) and PMRP (0.576 g/kg and 1.152 g/kg, Oral and Topical) (E) groups. The POMC, MITF, and ASIP contents in the skin tissue were measured by Elisa kits.

The $^{\#}$ indicates a significant difference compared with model group. $^{\#}p<0.05$; $^{\#\#}p<0.01$; $^{\#\#\#}p < 0.001$.

The * indicates a significant difference compared with normal group. $^{*}p<0.05$; $^{**}p<0.01$; $^{***}p < 0.001$.
